# The Rise and the Fall of Betatrophin/ANGPTL8 as an Inducer of *β*-Cell Proliferation

**DOI:** 10.1155/2016/4860595

**Published:** 2016-09-08

**Authors:** Mohamed Abu-Farha, Ashraf Al Madhoun, Jehad Abubaker

**Affiliations:** ^1^Biochemistry and Molecular Biology Unit, Dasman Diabetes Institute, Kuwait City, Kuwait; ^2^Research Division, Dasman Diabetes Institute, Kuwait City, Kuwait

## Abstract

Diabetes is a global health problem that is caused by impaired insulin production from pancreatic *β*-cells. Efforts to regenerate *β*-cells have been advancing rapidly in the past two decades with progress made towards identifying new agents that induce *β*-cells regeneration. ANGPTL8, also named betatrophin, has been recently identified as a hormone capable of inducing *β*-cells proliferation and increasing *β*-cells mass in rodents. Its discovery has been cherished as a breakthrough and a game changer in the field of *β*-cells regeneration. Initially, ANGPTL8 has been identified as atypical member of the angiopoietin-like protein family as a regulator of triglyceride in plasma through its interaction with ANGPTL3 and its regulation of lipoprotein lipase activity. In this review, we will review literature on the proposed role of ANGPTL8 in *β*-cells proliferation, the controversy regarding this role, and the emerging data questioning its involvement in *β*-cells proliferation. Additionally we will discuss new clinical data that describes its role in diabetes and the putative therapeutic targeting of this protein.

## 1. Introduction

In the past decade, diabetes has reached an epidemic stage affecting millions of people worldwide [1]. The majority of people are affected by type 2 diabetes (T2D) that is caused by impaired insulin secretion and/or insulin resistance that leads to improper blood glucose metabolism. Obesity associated insulin resistance is one of the main causes behind the progressive decline in insulin production by the pancreatic *β*-cells that ultimately leads to T2D [1–3]. Overall, insulin resistance results in increased hepatic glucose production, reduced muscle glucose uptake, and increased level of free fatty acids in plasma under fasting conditions amongst many more physiological changes [1, 3]. To combat insulin resistance, *β*-cells increase their insulin production to cope with the increased insulin demand before reaching a threshold, where they will not be able to cope with further increase in insulin demand. A myriad of factors are involved in the *β*-cells failure including aging, oxidative stress, genetic factors, lipotoxicity and glucotoxicity, and inflammation [1, 3]. Various types of medications are used to control plasma glucose level by targeting a number of biochemical pathways involved in decreasing hepatic glucose production, increasing insulin production, and increasing insulin sensitivity or direct insulin injection [1, 3]. In combination with lifestyle intervention these drugs can help diabetic patients reach a reasonable glycemic control; however, reaching a full glycemic control similar to healthy *β*-cells is very difficult to achieve [1, 2]. As a result, many studies are interested in identifying new and novel mechanisms to induce *β*-cell proliferation, which is valued as the ultimate treatment for both type 1 and type 2 diabetes. Therefore, numerous strategies and alternative cell sources have been utilized to generate *β*-like cells.

## 2. Beta-Cell Regeneration and the Identification of ANGPTL8

Current protocols for *β*-cell regeneration focus on the use of directed differentiation of embryonic or induced pluripotent stem cells into insulin producing cells. Proliferation of existing *β*-cells, reprograming nonpancreatic, or dedifferentiation of pancreatic non-beta-cells into beta-like cells approaches are also being experimented [4–7]. Β-cell mass maintenance is a dynamic process that keeps modifying depending on the metabolic demand throughout life. Β-cell proliferation rates progress during embryonic development, after that cells expansion declines at postnatal stage followed by gradual failing at maturity [4–7]. Interestingly, studies have detected an increase in *β*-cell mass during pregnancy and obesity [8–12], suggesting that new *β*-cells can form during adulthood. Yet, the main question remains about the mechanism that replenishes the *β*-cell reservoir; does it occur through preexisting beta-cells proliferation or through the existence of progenitor cells? Current studies have detected an increase in *β*-cell mitotic activity in response to pancreatic injury or experimental conditioned *β*-cells genetic ablation suggests that the replication process plays a central role in maintaining *β*-cell mass [13–17]. In human, *β*-cell neogenesis is not conclusive due to the fact that observations are drawn from pancreatic autopsy and surgical resection [18–20]. However, some experimental genetic lineage tracing and transgenic animal model approaches indicate that the progenitor cells are a subpopulation of the pancreatic duct epithelium [18] and/or centroacinar cells [21, 22]. On the other hand, new observations revealed that *β*-cell neogenesis is not decisive, implying the need for further investigations in the field [23–25]. On the other hand, in response to severe *β*-cell loss, interconversion of pancreatic endocrine cells has been reported in rodents [26, 27]. Several studies in rodents confirm that alteration in glucagon signaling enhanced alpha-cell regeneration, islets enlargement, and transdifferentiation into insulin producing cells [28, 29]. Nevertheless, this mechanism does not contribute to *β*-cell replenishment in diabetic animal models suggesting a significant role, but not limited, for glucagon in the transdifferentiation mechanism that is yet to be fully clarified [27, 30–32].

Overall, *β*-cell mass enrichment, through proliferation/neogenesis or interconversion, is regulated through a network of internal and external biochemical pathways. During the past several decades, studies were directed towards understanding the molecular mechanisms that influence *β*-cell mass. Several hormones were reported to amplify *β*-cell number including growth hormone [33], prolactin [34], placental lactogen [35], serotonin [36], glucagon like peptide-1 (GLP1) [37], insulin-like growth factor I (IGF-1) [38], and their prospective receptors, best reviewed in [39]. During pregnancy, the somatolactogenic hormones maintain normal glucose hemostasis through tyrosine signaling cascade activation, which causes a rise in intracellular Ca^2+^ and enhancement of insulin secretion [40]. On the other hand, GLP1 and IGF-1 increase cytosolic Ca^2+^ through protein kinase A (PKA) and mitogen-activated protein kinase (MAPK) signal transduction pathway, respectively [41, 42]. Recently, ghrelin has been reported to enhance *β*-cell mass [43], despite its antiapoptotic activity mediated by activated phosphatidylinositol 3-kinase (PI3K)/Akt and ERK1/2 signaling [44]. The epidermal growth factor betacellulin is also reported to mediate *β*-cell neogenesis through the activation of ErbB-1 and ErbB-2 receptors and the upregulation of IRS1 [45].

One of the most recently identified inducers of *β*-cell proliferation was a liver and adipose tissues secreted protein named betatrophin. It increases *β*-cell replication and *β*-cell mass in insulin resistance mouse model [49]. This hormone was identified after the authors injected mice with S961 peptide, an insulin receptor antagonist, generating an insulin resistance mouse model [49]. Using microarray technology the authors were able to identify genes that were upregulated as a result of this injection including betatrophin. Its overexpression was found to increase *β*-cell proliferation and mass [49]. Betatrophin is one of the names given to C19orf80, which is also called Hepatocellular Carcinoma-Associated Gene TD26, Refeeding Induced Fat and Liver (RIFL) [46], Lipasin [54], and ANGPTL8 [48]. ANGPTL8 will be mostly used hereafter in this review.

## 3. ANGPTL8 Role in Lipid Metabolism

Prior to its identification as a hormone involved in *β*-cell proliferation, ANGPTL8 was identified by a number of groups as a nutrient and heat regulated protein as well as a regulator of lipid metabolism [46, 48, 54, 55]. Ren et al. were one of the first to show that ANGPTL8 or RIFL, as they referred to it, was induced in 3T3-L1 cells during adipogenesis and its knockdown leads to reduction in adipogenesis [46]. A summary of selected studies is given in Table 1. ANGPTL8 effect on adipogenesis was also shown in primary mouse and human adipocytes [46]. They further looked at its transcript expression level in different mouse tissues showing its highest expression level in white and brown adipose tissues as well as the liver similar to what was later shown by the other groups [46, 48, 54, 55]. ANGPTL8 expression was induced in both adipose tissues and liver by feeding as well as insulin treatment [46]. Similarly, its level was higher in* ob/ob* obesity mouse model compared to wild type [46]. Quagliarini et al. gave this protein the name ANGPTL8 based on its sequence similarity to members of the angiopoietin-like protein family and showed that it interacted with ANGPTL3 and regulated TG plasma level in mice [48]. They also showed that a nonsynonymous SNP (R59W) was associated with lower LDL and HDL cholesterol without affecting the TG level [48]. We have recently reported that this variant was associated with increased fasting plasma glucose in an Arab population [56]. Concomitantly, Zhang showed that ANGPTL8 had sequence similarity to members of the angiopoietin-like protein family and referred to it as Lipasin due to its inhibition of lipoprotein lipase (LPL) activity [54]. Collectively, the previous studies demonstrated that ANGPTL8 was involved in regulating TG plasma level through its interaction with ANGPTL3 and inhibition of LPL activity [46, 48, 54, 55].

## 4. Role of ANGPTL8 in ***β***-Cell Proliferation

The identification of ANGPTL8 or betatrophin as a novel *β*-cell mitogen by Yi et al. has attracted tremendous attention from the scientific community as well as the media. It was hailed as a next-generation drug for diabetes. In their paper, Yi and his coworkers showed that S961 induced insulin resistance and was able to upregulate the expression of ANGPTL8 gene in the liver and adipose tissue. This upregulation of ANGPTL8 expression by insulin resistance was hypothesized to be a mechanism to increase insulin production through increasing *β*-cell proliferation. Overexpression of ANGPTL8 resulted in a 17-fold increase in *β*-cell proliferation and a threefold increase in *β*-cell mass [49]. As a result, mice overexpressing ANGPTL8 had improved glucose tolerance and lower fasting blood glucose [49]. ANGPTL8 level was also increased in* ob/ob* and* db/db* mouse models and during gestation in mice. Accordingly, it has been concluded by the authors that ANGPTL8 was capable of inducing *β*-cell mass and improving glucose tolerance and potentially augmenting or replacing insulin injections [49]. Even though the data was very promising, further validation such as testing the regenerative effect of betatrophin on aged and diabetic mice and human studies demonstrating the beneficial effect of ANGPTL8 on human pancreatic *β*-cells are deemed necessary [57, 58].

These conclusions were questioned by other studies that showed mice lacking ANGPTL8 had normal glucose and insulin tolerance [59]. Using ANGPTL8 knockout mouse model, Wang et al. showed that ANGPTL8 was required to direct free fatty acid into adipose tissue for storage after food intake through regulating the activity of LPL. However, lack of ANGPTL8 did not affect glucose and insulin tolerance and did not show significant changes in glucose homeostasis [59]. Even though their data failed to show a role for ANGPTL8 in glucose homeostasis they did not rule out the possibility that supraphysiological concentrations of ANGPTL8 might be able to induce *β*-cell proliferation at the potential cost of inducing hypertriglyceridemia [59].

In order to test the effect of ANGPTL8 on human *β*-cells, Jiao et al. treated immune-deficient NOD-Scid mice with S961 to induce insulin resistance and ANGPTL8 expression [60]. Treating these mice with S961 resulted in a significant increase in ANGPTL8 expression as well as *β*-cell replication in the native as well as the ectopically transplanted mice islets under the kidney capsule. However, treatment did not cause any increase in *β*-cell proliferation using human transplanted islets [60]. Even though they did not address whether mouse produced ANGPTL8 was capable of binding to its unidentified receptor on the human *β*-cells, they showed that the increased ANGPTL8 level was not capable of inducing *β*-cell proliferation in humans [60]. Furthermore, Gusarova et al. tested the effect of ANGPTL8 knockout on *β*-cell proliferation and reported that *β*-cell proliferation was not affected by the lack of ANGPTL8 in response to diet induced insulin resistance or the S961 insulin receptor antagonist treatment. They also showed that increased ANGPTL8 expression did not increase *β*-cell mass or improved glucose homeostasis [50]. However, they further confirmed that TG level was reduced in knockout mice and increased by ANGPTL8 overexpression [50]. Later on, Yi et al. have showed that they were not able to reproduce their original data and cited huge variation of the effect of ANGPTL8 injection on *β*-cell proliferation. In conclusion, it has been shown that ANGPTL8 induction of *β*-cell proliferation in mice was not reproducible and its deletion did not affect *β*-cell proliferation as suggested earlier. These reports raise major concerns regarding new inducers of *β*-cell proliferation and ask for more stringent measures to ensure their accuracy. Some of these measures include testing these mitogenic substances on human islets to test for *β*-cell proliferation. Additionally, therapeutically relevant levels of human *β*-cell proliferation ought to be achieved before rushing into conclusions as previously suggested [61].

## 5. ANGPTL8 Role in Diabetes

Irrespective of its role in *β*-cell proliferation, initial studies on ANGPTL8 reported that it was induced by insulin [46, 49]. Other human studies also showed that ANGPTL8 was positively associated with insulin [52, 62–64]. On the other hand, its plasma level in diabetes has been measured in multiple cohorts [51–53, 62, 64–72]. Initial mice studies showed that ANGPTL8 level was increased in* ob/ob* mice as well as the diabetic mouse model* db/db* [49]. In humans, Espes et al. showed that circulation level of ANGPTL8 was increased in T1D subjects [51]. Nonetheless, ANGPTL8 level did not correlate with an increase in C-peptide level in T1D [51]. Similarly, in another study, Espes et al. also showed that ANGPTL8 level was increased in T2D subjects. Other studies showed that ANGPTL8 level was increased in T2D as well [64, 67, 68, 70].

Using a large sample set of T2D and normal subjects, we have recently reported that ANGPTL8 was increased in T2D subjects [52]. Comparing the level of ANGPTL8 in 556 T2D subjects with that of 1047 nondiabetic subjects we showed that ANGPTL8 level was more than three times higher in T2D subjects [52]. ANGPTL8 level was associated with blood glucose, insulin, and insulin resistance as measured by homeostatic model assessment-insulin resistance (HOMA-IR) in the nondiabetic subjects only. No association was observed with these factors in the T2D subjects [52]. Furthermore, we have showed that ANGPTL8 level was associated with increased C-peptide level in the nondiabetic subjects but not the T2D subjects. Taken together, our data revealed that the increase in ANGPTL8 in T2D was not increasing insulin production in the T2D subjects [62]. On the other hand, other studies showed that ANGPTL8 was not increased in T2D subject and rather decreased [53, 63]. Recently, Li et al. have published a meta-analysis that investigated the association between ANGPTL8 level and T2D based on a total of nine studies [73]. Based on their analysis, ANGPTL8 level was significantly higher in subjects with T2D compared to nondiabetics [73]. Similarly, ANGPTL8 expression level was increased in subjects with gestational diabetes [74–77]. ANGPTL8 level was also found to be increased in subjects with metabolic syndrome [71]. One of the main causes for differences in these results has been suggested to be the ELISA kits used. Fu et al. tested both full length ELISA kit from Wuhan EIAAB Science Co. (catalogue number E1164H) and C-terminal ELISA kit recognizing the region from 139 to 198 amino acids from Phoenix Pharmaceuticals (catalogue number EK-051-55) [78]. Both kits were found to be accurate at measuring plasma level of ANGPTL8 and showed high correlation [78]. However, the studies showing decreased plasma level in diabetes used a third kit from ELISA kit manufactured by CUSABIO/Aviscera Bioscience which might have been the cause for the discrepancy [53, 63].

## 6. Conclusion

Overall, ANGPTL8 role in lipid metabolism is well established, through its interaction with ANGPTL3 and regulation of LPL activity to maintain blood TG content. It is also well established that ANGPTL8 does not play a major role in *β*-cell proliferation as proposed initially. Its role in diabetes and obesity however remains elusive and further studies are still required to understand its involvement in metabolic diseases. New data is emerging to link ANGPTL8 to other diseases such as cancer and polycystic ovary syndrome [79] will increase our knowledge of the functional role of this hormone. In conclusion, *β*-cell regeneration remains an ultimate goal in diabetes treatment and detailed understanding of their biology and agents that manipulate their function is required. Nonetheless, human studies as well as more rigorous experimental design are required to test for new agents associated with *β*-cell regeneration to avoid further setbacks similar to what has been observed with betatrophin.

## Figures and Tables

**Table 1:** Selected early studies investigating the role of ANGPTL8 in obesity and diabetes.

Study design	Findings	Refs
Study was designed to identify new genes involved in lipid metabolism based on Lexicon-Genentech knockout database of genes that was generated for 3T3-L1 in vitro adipogenesis.	One of the first reports to study the role of ANGPTL8 or RIFL as they called it in lipid metabolism and highlighted its role in adipocyte differentiation and its similarity to ANGPTL3. They also showed that RIFL was induced by insulin.	[46]

Identification of lipid metabolism genes using transcriptomic analysis on liver and fat tissues extracted from mice treated with a high-fat diet or fasting using RNA-seq experiments.	ANGPTL8 or Lipasin was identified as a nutritionally regulated protein produced by the liver that regulates plasma lipid contents by affecting lipoprotein lipase activity.	[47]

Investigation of the ANGPTL8 role in lipid metabolism and identification of its variant in humans.	Identification of ANGPTL8 as a regulator of triglyceride in plasma through its interaction with ANGPTL3 that regulates the activity of lipoprotein lipase activity. They also identified an ANGPTL8 variant that was associated with reduced LDL and HDL levels.	[48]

Identification of insulin resistance related genes by inducing insulin resistance via treatment with S961 insulin receptor antagonist.	Identification of betatrophin as insulin resistance induced genes in the liver and white and brown adipose tissues in mice and humans. Its expression was increased over threefold in the liver of both *ob/ob* and *db/db* mice. It was also demonstrated to increase beta-cell proliferation and mass.	[49]

Use ANGPTL8 knockout model to investigate the role of this protein in beta-cell proliferation and glucose metabolism.	Based on their data they concluded that ANGPTL8 was not involved in controlling beta-cell growth in mice unlike what has been previously reported by Yi et al. [49].	[50]

Study the plasma level of ANGPTL8 in 33 people with T1D and their controls.	ANGPTL8 was increased in subjects with T1D.	[51]

Investigate changes in the level of ANGPTL8 in a large cohort of 1049 nondiabetic people and 556 people with T2D.	Increased ANGPTL8 in obese and T2D people. ANGPTL8 was positively associated with fasting blood glucose, HOMA-IR, and duration of diabetes.	[52]

Compare plasma level of ANGPTL8 in normal people with various glycemic indices as well as T2D people with their proper controls.	One of the only studies to show that ANGPTL8 level was decreased in obese people as well as people with T2D. This study used a different ELISA kit to measure plasma level of ANGPTL8 than what has been used by the other studies.	[53].
